# The Effects of Housing on Health and Health Risks in an Aging Population: A Qualitative Study in Rural Thailand

**DOI:** 10.1155/2014/289731

**Published:** 2014-07-02

**Authors:** Ratana Somrongthong, Saovalux Dullyaperadis, Anne Louise Wulff, Paul R. Ward

**Affiliations:** ^1^College of Public Health Sciences, Chulalongkorn University, Bangkok 10330, Thailand; ^2^School of Health Promotion, Planning and Strategy, Roskilde University, 4000 Roskilde, Denmark; ^3^Discipline of Public Health, Flinders University, Adelaide 5001, Australia

## Abstract

*Background*. Over the last decade, Thailand has experienced an aging population, especially in rural areas. Research finds a strong, positive relationship between good quality housing and health, and this paper assesses the impact and living experience of housing of older people in rural Thailand. *Methods*. This was a mixed-method study, using data from observations of the physical adequacy of housing, semistructured interviews with key informants, and archival information from health records for 13 households in rural Thailand. *Results*. There were four main themes, each of which led to health risks for the older people: “lighting and unsafe wires,” “house design and composition,” “maintenance of the house,” and “health care equipment.” The housing was not appropriately designed to accommodate health care equipment or to fully support individual daily activities of older people. Numerous accidents occurred as a direct result of inadequate housing and the majority of houses had insufficient and unsafe lighting, floor surfaces and furniture that created health risks, and toilets or beds that were at an unsuitable height for older people. *Conclusion*. This paper provides an improved and an important understanding of the housing situation among older people living in rural areas in Thailand.

## 1. Introduction

In Thailand, as in many developing countries, there has been a decline in birthrates and a rising life expectancy, which together have led to a demographic transformation. In many developed and developing countries, the total fertility rate is already estimated to be beneath the replacement level [1, 2]. This global phenomenon has led, among other things, to an increased relative number of people aged 60 years and above [2–4]. By the year 2050, it is estimated that 80% of these older people will live in developing countries, where the growth rate is progressing twice as fast compared with the industrialized countries [5, 6]. Presently, Asia has the highest proportion of elderly people, which is also expected to be so in the future. The World Health Organization predicts that, by 2050, one in four Asians will be older than 60 years [7–9].

On one level, one may herald this increase in older people as a global success since it is underpinned by reduced mortality rates. However, whilst the majority of older people will be living in developing Asian countries, it is these countries that are often least prepared to meet the challenges of an aging population [10, 11]. The majority of government expenditure on health and social care is still focused on fighting communicable diseases and dealing with the acute response to emerging noncommunicable diseases such as diabetes, coronary heart disease, and many cancers. This is certainly the case in Thailand, which is ranked as the second most aged country in Asia [10, 12]. We do not underestimate the policy problem for governments but argue that we are likely to have an increased problem for older people, especially in rural areas, if we do not deal with their immediate needs, one of which is adequate housing conditions to sustain health and prevent illness.

Thailand has witnessed an increase in life expectancy and increased quality of life for older people [12]. However, whilst a large proportion of older people in Thailand are healthy enough to take care of themselves, there are an increasing number of older people who have reduced quality of life and have difficulties in undertaking essential daily activities [13]. There has also been an increasing prevalence of noncommunicable diseases in Thailand, such as diabetes, osteoarthritis, cardio-cerebrovascular diseases, and cancer, which compound problems for older people [13, 14].

In rural areas of Thailand, the majority of houses are made from wood and are built on stilts, about 1 to 1.5 metres off the ground. The main benefits of this are to keep the house above flood waters and to keep air circulating around the house in the hot climate. The space below the house also provides a useful storage area or a place where domestic animals can be kept. In rural areas, the majority of work revolves around agriculture. Village families work together, often sharing tools and equipment, to plant and harvest their crops (rice, corn, taro, etc.) and to raise their animals. After feeding their families, any extra food produced is sold at a local market, where clothing, cooking oil, and utensils can be bought. Some of the village men work as builders and carpenters, and others travel to factories nearby or work on plantations which grow rubber, tea, or coffee. The village people share religious and cultural celebrations, gathering together for weddings and festivals. Older people tend to live with or nearby their adult children, who are expected to perform caring roles [15]. The location for this study, Saraburi province, has all of the attributes listed above—a very typical Thai rural location.

Although ageing is a triumph of development, it also poses many challenges to the society and the surrounding environment. Increased age normally brings changes in health and social needs, so, for example, redesign of the houses where the older people live is necessary for maintaining favorable health conditions and supporting health promoting behaviors [3, 16, 17]. Numerous studies suggest a direct relationship between health and housing, particularly when it comes to older people [18–20]. Indeed, the World Health Organization has implied for a long time that an environment that supports healthy living and well-being is an important goal in health promotion, planning, and strategies [21].

Policy is required to reduce unsafe housing in rural Thailand, especially for older and frailer people. Improving the safety of houses may help to compensate for limitations and decline in functional abilities and also help to maintain health promoting conditions. The physical environment, if constructed and designed well, can enable older people to carry out basic independent activities associated with daily living. In addition, there are numerous risks and hazards within the physical environment, especially in rural areas, which may pose serious risks to health. Furthermore the physical environment can support the provision of health care services [18, 20, 22]. According to studies from the US and Europe, many households are not designed in a way that enhances or maintains older people's health [17, 23, 24]. However, less is known about the physical environment of older people in developing countries, particularly in resource-limited areas like Thailand [7, 10, 12, 25]. At present, no published study has reported the physical barriers in households of older people living in rural areas in developing countries or the perceptions of older people in terms of the risks to their health posed by inadequate housing.

This paper presents data from a qualitative study with older people (over 60 years) in rural Thailand, which aimed to explore the effects of housing and the physical environment on their health. The key purpose of this paper is to highlight the health risks posed by housing for older people in rural areas, which is likely to be an increasing problem given the demographic shifts in Thailand and other developing countries. Whilst we recognize the potential difficulties in shifting policy and resources to improve housing stock in rural areas in developing countries, we need to raise the issue as one which is worthy of policy response.

## 2. Methods

This study used a range of complimentary methods and data sources to address the aims of the study. The data were obtained by observations of the physical adequacy of the housing, semistructured interviews with a variety of key informants, and archival information in the form of health records in order to assess any healthcare utilization related to poor housing.

The observation of the physical adequacy of the housing was undertaken by two trained researchers who walked around the houses making notes on all issues that they determined to have the potential of having an impact on health. Based on previous observations of houses in rural areas of Thailand, an observation checklist was developed and pilot tested prior to the observations. This checklist was divided into sections, which itemized the things to look for in the houses, such as faulty electrics, the position and height of furniture in terms of difficulty getting into or out of chairs and beds, and any modifications that help or hinder the older person's movement around the house. The checklist was designed so that it could be used for evaluating whether the housing of the older person was structured in a manner that promotes, supports, and maintains their health. In each section, the observers could answer “yes,” “no,” or “not clear.” The answers were based on observations and on the responses obtained from questioning the older people themselves. An example of a question could be as follows: “is the furniture age-appropriate in a way that has potential for supporting healthy living?” All observations were undertaken by two independent observers in order to minimize observer bias.

The semistructured interviews were conducted continuously during the home visits and at the local health promoting hospital. The main purposes of these interviews were to support, validate, clarify, and evaluate the findings from the observations. Furthermore, the interviews gave a deeper understanding of the way of living in the villages and, moreover, an understanding of the health issues that were related to the physical environment. In order to provide both depth and context within the study, we interviewed four different kinds of key informants. Firstly, we interviewed the older people who lived in the houses in order to get first-hand experiences and perceptions of the adequacy of their housing, perceptions of any risks to their health, and experiences of the ways in which their physical environment may have led to accidents or illness. Secondly, we interviewed the “family caretakers” of the older people, who were generally younger relatives who lived either with them or nearby (less than 100 metres). These interviews focused on their perceptions of the housing and the risks posed to their older relatives. Thirdly, we interviewed local health volunteers who assist the older people with a variety of health and social care needs. Whilst not being family members, these people have close knowledge of the older people, their houses and illness, or accidents they have had in relation to their physical environment. Finally, we interviewed staff from the local health promoting hospital to get their broader perspectives on the impact of housing and the physical environment on health, illness, and accidents of older people within their area.

Moreover, archival information, in the form of health and medical records, was used to fill in arising gaps, such as history of illnesses or accidents that might be associated with the physical environment of the households.

## 3. Setting and Participants

The participants were purposively recruited from two different subdistricts, Taladnoy and Horathep, which are both within the same province in Thailand. The characteristics of the two areas are rural settings with a large number of elderly residents. These two locations were purposively chosen because they represent rural areas with an aging population.

A final sample size of 47 key informants (semistructured interviews) and 13 households (observation of housing adequacy) was generated within this study, which we believe contributed to a sufficient, adequate, and saturated set of data. Numbers instead of names were used to keep track of the households, in order to avoid any identification details in contravention of our research ethics procedures.

The households used for investigation were obtained from a list, containing an overview of all households in the two subdistricts, provided by the Chief of the local health promoting hospital in Saraburi Province. A total of 15 households were selected and initially visited. The inclusion criteria for the study were being 60 years of age and above; living for at least one year in Taladnoy and Horathep subdistricts in Saraburi Province; and being able to understand and communicate sufficiently in Thai. Exclusion criteria included not being at home at the time of the visits or having a current serious medical or psychiatric illness. Of the 15 households, two were ineligible: one due to not meeting the inclusion criteria and one due to the older person declining participation. The remaining 13 households all agreed to participate in this study. This number of participants was consistent with other similar studies in rural areas [26]. Furthermore, this number was found to provide saturated information on the basis of the research question.

The participants were provided written or verbal informed consent on the first visit. They were all informed about the aim and the use of this study and assured their anonymity. Any information that might identify an individual participant was not recorded. Within this paper, we identify specific quotes from interviews with simply the age and gender of the participant. This study was approved by The Ethics Review Committee for Research Involving Human Research Subjects, Health Science Group, at Chulalongkorn University.

## 4. Data Analysis

All interviews were audio-recorded and transcribed in Thai. Following transcription and checking for accuracy, interview data were analysed using two forms of analysis: open coding and focused coding [27, 28]. Our open coding provided a description of the issues or themes arising from the data. This was undertaken throughout the data collection process, and our initial open coding formed the content of subsequent interviews. Each interview was transcribed in Thai directly after the interview so that the data analysis and collection could be compared. The process of open coding followed the following recognised stages: breaking down, examining, comparing, conceptualising, and categorising data [29]. In the process of open coding, words or sections of text were coded using the actual words used by participants, or by grouping similar words conceptually. Focused coding was undertaken by grouping the open codes into larger categories. This process involved an iterative process of slotting each of the initial open codes into larger categories, based on the ways in which they seemed to be relating to a similar idea or issue. These initial focused codes were quite large and needed to be reduced over a number of analytical readings of the codes in order to permit sensible interpretation.

## 5. Results

### 5.1. Characteristics of the Study Participants

During this study, 13 households were visited and 47 key informants were interviewed. Older couples inhabited three of the 13 households, and in these households, interviews were undertaken with both partners. However, none of the older people participating in this study were living entirely by themselves. 11 of the households were cohabited with the families of the older people. In the last two households, the families of the older people were living in separate houses, but on the same property a short distance away.

Of the 16 interviews with older people, we had seven men and nine women, and 13 older people were aged under 75 years (age range 63–96 years). All of the older people had lived in that particular house for more than 10 years and none of them expressed a wish to move. The family caretakers of the older people were all closely related, being either sisters, daughters, sons, grandchildren, or brothers/sisters in-law. The family caretakers' ages ranged from 21–67 years.

The village health volunteers interviewed were mainly female (10/11), ranging in age from 39–57 years. Seven of them had worked voluntarily for the health service for approximately 10 years, though the range of experiences varied from three to twenty years. All of them worked voluntarily three days per week, six hours per day. During a normal workday, the volunteers visit six to eight households, with duration of 30 minutes per visit.

The three staff members interviewed from the local health promoting hospital included two women and one man, all nurses in their 30s. Their range of experiences varied from two to six years. This local health promoting hospital was in the area of the households observed and was the preferred place to go for people with health issues. Furthermore, the health promoting hospital closely cooperated with the village health volunteers, who report back to the staff members after every home visit.

The findings from this study were categorized into four main focused codes. These focused codes were based on the most recurrent and pertinent focused codes related to the physical environment, in order to answer the research objectives. The themes were “lighting and unsafe wires,” “house design and composition,” “maintenance of the house,” and “health care equipment.” In describing these focused codes, we focus specifically on their impact on health, illness, accidents, and health risks.

### 5.2. Lighting and Unsafe Wires

Among the 13 households, 10 had insufficient lightning, characterized by a lack of proper or functional lighting in the entire house or in part of the house and inappropriate placed outlets, wires, and lamps. In nine of these households, the older people reported that they could not reach the power outlets that the lamps were connected to, which therefore made them dependent on other people turning on/off the electricity. For example, “*even to turn on the light can be a problem in my age*” (female, 74 years old).

Oil lamps were used occasionally in five of the households, which the older people could not turn on/off themselves due to their declining mobility. The wires connected to the lamps were observed crossing the floor or placed in other unsuitable ways in 12 of the households. These were observed as hazards, and the older people talked about how they presented a barrier for them when it came to moving easily around, thereby reducing their mobility and potential for physical activity. These findings were also supported and confirmed by the key informants including family members and health volunteers, who also provided examples of when the loose cables had resulted in accidents, such as falls, for the older people. They also talked about how the accidents get worse during power failure, which often happens due to heavy rain or/and flooding, since the older people cannot see the loose wires and cables. Furthermore, the archival information of medical records revealed that all of the older people visited had declining vision, which makes good lighting even more essential in order to maintain independent behavior, the potential for physical activity and also for meeting neighbors. Whilst meeting neighbors may, on the face of it, not seem of huge importance, research in Thailand and elsewhere shows the critical importance of such interpersonal relationships for the development of social capital and mental health promotion [30, 31].

### 5.3. House Design and Composition

This study also found that the compositions of the households were not always designed to accommodate optimal assistance from caregivers to the older people. In addition, the poor design of the houses also hindered the functioning daily activities of the older people, including relatively straightforward activities like getting into/out of bed, getting on/off the toilet, and walking within the house. In all households, partly missing hand rails at the stairs were observed, which was recognized by the older people as a barrier for walking up and down the stairs and often meant they were confined to the ground floor of the house.

One of the most conspicuous findings was the low height of the beds and toilets, which we observed in 11 of the 13 households. The family members and older people in these households stated that they had a hard time maneuvering up and down from either the toilets or beds. Due to the height of the toilet, almost six of the older people were forced to use other ways to relieve themselves, reducing the sense of autonomy, dignity, and agency. Moreover, 9 older people confirmed that they were dependent on other people to help them get in and out of bed or go to the toilet. For example, “*I wait for my family to come and help me getting out of bed every morning*” (male, 93 years old).

Nine of the households had high doorsteps at the entrance to the house (more than 10 cm), which the older people reported as being either hard to step over or not possible at all. For example, one person said, “*I'm almost locked inside this room, because of this* (pointing at the door-step)” (female, age 73 years). The allocation of the rooms in the houses also led to some obstruction and barriers. In 11 households, the kitchen and the toilet were outside the house, which all the older people reported as a major barrier for them in terms of them relying on others to prepare and cook their food and also having to wait for others for their toilet needs. In addition to impacting their functional needs, these design issues also impacted their dignity and sense of self, since the older people used to delight in cooking food for themselves and their families, but are no longer able to do so. In addition, all of the households had their bedrooms on the 1st floor; however, only seven of the older people were still able to make it to the 1st floor, due to functional disabilities, and therefore able to sleep in their original bedrooms. The other nine older people had to sleep downstairs. An older woman aged 85 years confirmed this by saying: “*It has been many years since I have seen the 1st floor at my own house.*”

### 5.4. Maintenance of the House

In addition to the design and composition of the households, several factors associated with the maintenance of the houses were also observed as barriers to the physical and mental health of the older people. One of the major problems observed in 11 of the houses was the partly damaged wooden or concrete floor inside the houses, which often had uneven surfaces, which made it difficult for the older people to walk safely. In addition to our observation of the unsafe floors, the older people, family members, and health volunteers all talked about the risk of the floor in terms of accidents, which was borne out in our analysis of the medical records which showed several accidents for the older participants. Older people themselves talked about their decrease in safe and independent functioning of daily activities as a result of the unsafe floors. For example, one person said, “*I'm very scared of falling. I have already done it more than once, because of the floor*” (male, 71 years old).

A further physical barrier was also observed within the decoration of the floors. In seven households, there were either plastic or carpets covering part of the floor, which could be unsafe to walk on and increase the fall hazard among the older people. This is especially the case in countries like Thailand, where the culture is to walk around barefoot inside the houses.

The furniture in seven of the households was observed to be partly damaged, such as broken armchairs or missing chair backs, which has resulted in less than optimal living conditions and may result in further accidents or immobility. Most of the households visited were also observed to have numerous of chairs without armrests, which often, confirmed by the older people interviewed, resulted in a lack of desire for using them, due to difficulties of getting up again.

In addition, we observed a lack of space for storage in nine houses, which resulted in food, water containers, and garbage taking up large amounts of space inside the house. In reporting this, we do not wish to sully the identities of the participants in our study, since they had little choice for storage. In addition to the potential impact on health of keeping garbage in the house, it also reduced the available space for safe walking. This was also confirmed in the interviews with family members, caretakers, and older people, who reported a lack of sufficient space and layout for safe walking and mobility, assistive technologies, and other health care equipment or for personal assistance. For example, “*With all these things stored inside the houses it's not possible to help the way we want*” (village health volunteer, female).

### 5.5. Health Care Equipment

Another main finding of this study was the deficient number of health care equipment required for healthy functioning, such as handgrips, handrails, wheel chairs, toilet-chairs, and walkers. These health care pieces of equipment could compensate for limitations in the functional abilities of the older people and could enable them to carry out basic activities of daily living, but were not present in households. Our knowledge of the local health care system is that such equipment is available for older people, but it was not observed in seven of the households. However, in these households that did not have the health care equipment, alternative tools and instruments had been made, such as wooden sticks to walk with or ropes to hold on to when navigating around the house. Another interesting finding was that although six of the households had one or more of the health care technologies, they were not necessarily used by the older people. Indeed, in four households where health care technologies were observed, they were actually stored away, out of the reach of the older people, rendering them useless.

## 6. Discussion

### 6.1. Limitations and Strengths of the Study

In this study, 13 purposely selected households within the same province were observed, which could limit any external validity and generalizability of our findings. However, the 13 households in this study were found to match a national cross section of older people in Thailand, based on age and gender diversity. Nine out of the 16 older people were women, which closely resembles the Thailand national distribution of 56% [15]. Nevertheless, in order to generalize the findings to other areas, researchers need to apply our methods with a larger number of households, possibly randomly sampled.

One of the great strengths of this study was the objective nature of the household observations, undertaken by two independent researchers using the same checklist. If the researchers did not agree on a particular item, then the data were rejected. Additionally, multiple methods for gathering the data were used. By using multiple methods (archival, informal interviews, and observations), the information gained through the observations was corroborated, similar to the process of triangulation.

### 6.2. Key Findings

This study shows that the housing of older people in rural areas in Thailand is not appropriately designed to accommodate health care equipment and health care assistance from caregivers as needed or to fully support individual daily activities of older people, in order to facilitate proper health maintenance or promote health behaviors. There are several physical barriers within the households of the older people that pose challenges to the individuals and their families. However, it is important to be aware that these households are in rural areas and therefore characterized by residents who often have limited resources. Nevertheless, as humans, they have the same rights as others, and therefore may benefit from a policy designed to improve their living conditions, especially given the increase in the proportion of the population composed of older people, both now and in the future.

We acknowledge that the physical environment on its own does not provide the basis for a healthy living, since individuals have agency and choice. Nevertheless, the physical environment needs to be part of the constellation of factors affecting policy on the social determinants of health, alongside an understanding of the psychological, social, and political environments. The results found in this study should, therefore, not be used in isolation as a basis for developing interventions or judgments about the older people or their families, but as an important and necessary knowledge base to understand and improve the activity, health, and health care of older people in rural areas.

Interestingly, whilst we observed inadequate and unsafe housing and the analysis of medical records showed numerous accidents as a direct result of this housing, none of the older people complained or reported that they felt unhappy or left on their own. Rather, the older people all appeared satisfied and pleased with their situation, although they often qualified this by suggesting that their age needs to be taken into consideration, which is a form of normalization. Therefore, simply asking older people about their healthcare and housing needs may not be sufficient. Our method of objectively observing the housing conditions may be required in order to reduce the impact of normalization on future changes to housing.

A previous study found that 10% of older people in Thailand experienced a fall during the previous 6 months, and 45% of these experienced more than one fall. Almost half of falls occurred inside the house [32]. Our study found several problems with housing that could influence the mobility and activity levels of the older people. However, it is unknown whether the activity level of these people would improve as a result of restructuring and redesigning the houses. This needs to be examined in future intervention studies in rural areas, especially given the potential for culturally entrenched views and practices in older people to act as a barrier to change. Nevertheless, numerous other studies have found that the physical environment, if constructed and designed well, can enable older people to carry out basic independent activities associated with daily living and to maintain a preferred activity level [18, 20, 32]. Furthermore, homes have become a growing preferred environment for health care delivery, both in developed and developing countries. Several studies, from Europe and the US, have addressed this upcoming trend of health care delivery and found that a more supportive and adequate physical environment can reduce the needs for health care and increase the well-being [17, 20, 23, 33]. When interpreting the results from our study, it is also important to take into account that several of the physical barriers that were observed were, to a large extent, met and solved by family members and health volunteers. All of the households visited during this study received daily help from their relatives, who were living either together with them or nearby. Although the family structures within the households might help overcome some physical barriers, the findings from this study are still important at a time when developing countries are in transition and show signs of moving toward Western-style patterns of social structure, whereby health and social and welfare systems are provided by organizations and institutions outside the family.

In 2001, only 4% of older people in Thailand received a pension from the Government and a further 3% received welfare payments [10]. Almost 75% of financial support for older people in Thailand came from paid employment and/or their families. In order to respond to this, in 2009, Thailand implemented a large-scale expansion of its tax-financed “social” pension. While the scheme had previously been targeted to the poor, in 2009 it was extended to all those who were not receiving another form of government “formal” pension, thereby establishing universal pension coverage for all over 60s. Currently, the pension scheme provides beneficiaries of between 600 THB and 1,000 THB per month (approximately US$19 to US$32), with transfers increasing by age. Older people in Thailand (over 60 years) also have access to free and universal healthcare services, enshrined by the constitution [13]. However, a number of older people in rural areas find it difficult to access healthcare services, especially government hospitals, due to the long distances involved [13].

Our data provides an improved and important understanding of the housing situation among older people living in rural areas in Thailand. The results indicate that developing countries, like Thailand, could face an increase in the burdens related to the households of older people in the near future. The physical barriers, found in this study, need to be addressed and improved in order to prepare the societies for an aging population. However, the success of the households as a health-supporting environment should be assessed and understood in a more complex manner than simply modifying the physical environment of the households—they need to be able to enhance physical activity, psychosocial health, and broader physical and mental health care needs. Furthermore, studies are needed to examine to what extent the physical issues and concerns are perceived as “barriers” by older people themselves. Finally, the applicability of this study to other countries remains to be assessed.
